# Conservation of Markers and Stemness in Adipose Stem and Progenitor Cells between Cattle and Other Species

**DOI:** 10.3390/ijms241511908

**Published:** 2023-07-25

**Authors:** Yuki Ishida, Yo Mabuchi, Yuna Naraoka, Daisuke Hisamatsu, Chihiro Akazawa

**Affiliations:** 1 Intractable Disease Research Center, Juntendo University Graduate School of Medicine, 2-1-1, Hongo, Bunkyo-ku, Tokyo 113-8421, Japan; y.ishida.ix@juntendo.ac.jp (Y.I.); y.mabuchi.jy@juntendo.ac.jp (Y.M.); ynaraoka@juntendo.ac.jp (Y.N.); d.hisamatsu.ap@juntendo.ac.jp (D.H.); 2 Department of Clinical Regenerative Medicine, Fujita Health University, Toyoake 470-1192, Japan

**Keywords:** adipose stem and progenitor cells, adipogenic stem cells, preadipocytes, bovine, conservation

## Abstract

Adipose stem and progenitor cells (ASPCs) have been isolated from humans and animals for use in regenerative medicine and therapy. However, knowledge of ASPCs in other species is limited. Particularly, ASPCs in livestock are expected to enhance the fat content and meat composition. In this study, we isolated bovine ASPCs using cell surface markers. Specifically, we focused on ASPC markers in humans and experimental animals, namely CD26, CD146, and CD54. Stromal vascular fraction cells from bovine fat were separated using flow cytometry before primary culture. We evaluated the self-renewal and adipogenic potential of each fraction. We identified four cell populations: CD26−CD146+CD54+, CD26−CD146+CD54−, CD26−CD146−, and CD26+CD146−. Among them, the CD26−CD146+ fraction, particularly CD54+, demonstrated the properties of preadipocytes (PreAs), characterized by slow proliferation and a high adipogenic capacity. In conclusion, we could collect and characterize possible PreAs as CD26−CD146+CD54+ or CD26−CD146+CD54−, which are expected for in vitro bovine adipogenic assays in the future.

## 1. Introduction

Adipose stem and progenitor cells (ASPCs) are stromal vascular fraction (SVF) cells derived from adipose tissue and do not include immune, endothelial, or red blood cells [1]. These cells are heterogeneous, and several studies have aimed to characterize and classify them in humans and experimental animals such as mice. Particularly, researchers have targeted cell surface markers to analyze and isolate distinctive cells. Compared to humans and experimental animals, there have been few reports on ASPCs in other species, despite their importance. ASPCs in livestock, in particular, are expected to enhance the fat content and composition in food through in vitro assays of fatty acid metabolism, facilitation of lipogenesis and production of specific fatty acids. SVF cells derived from bovine fat have been reported to exhibit properties resembling mesenchymal stem/stromal cells (MSCs), such as a fibroblast-like morphology and multilineage differentiation [2]. However, further classification of ASPCs in bovine fat is yet to be conducted. Moreover, MSCs from different species cannot be identified or isolated using the same markers [3].

Lin−CD29+CD34+Sca-1+CD24+ cells in the white adipose tissue (WAT) of adult mice have been reported to be adipocyte progenitor cells and can reconstruct functional white adipose tissue in vivo [4]. Moreover, single-cell RNA sequencing (scRNA-seq) has made it possible to classify clusters based on gene expression and identify specific cell surface markers. For example, CD142+ cells in the subcutaneous and visceral adipose tissues of mice were found to inhibit adipogenesis via a paracrine mechanism and were named adipogenesis regulatory cells (Aregs) [5]. In addition, CD54+ cells in human and mouse WAT have been reported to be committed preadipocytes that differentiate into mature adipocytes with minimal stimulation [6]. Furthermore, CD26+ cells are highly proliferative and multipotent progenitors capable of differentiating into CD54+ and CD142+ cells [6]. Ferrero et al. reviewed the heterogeneity of ASPCs throughout previous works and classified them into “adipogenic stem cells (ASCs): CD26+”, “preadipocytes (PreAs): CD54+”, or “adipogenesis regulators (Aregs): CD142+” [1].

The mechanisms underlying adipogenesis in ASPCs, particularly those associated with cell surface markers, have been investigated. CD146 (a melanoma cell adhesion molecule, MCAM) has been identified as a ligand that interacts with more than 10 molecules, including components of the extracellular matrix, proangiogenic factor receptors, and growth factors [7]. Among these, angiopoietin-like protein 2 (ANGPTL2) binds to CD146 and facilitates adipogenesis, lipid accumulation, and adipose inflammation through the activation of cAMP response element-binding protein [8]. Consistent with this report, human CD146+ cells are highly adipogenic stem and progenitor cells [9,10]. However, it is unclear whether bovine CD146+ cells share this capacity.

In this study, we isolated novel ASPC populations based on the expression patterns of cell surface markers and confirmed whether the markers and stemness of human and mouse ASPCs, such as ASCs, PreAs, and Aregs, are conserved in cattle. Specifically, we performed prospective isolation using flow cytometry before primary culture. We then evaluated the self-renewal and adipogenic capacities of the isolated ASPCs.

## 2. Results

### 2.1. Isolation and Classification of ASPCs

**Figure 1:**
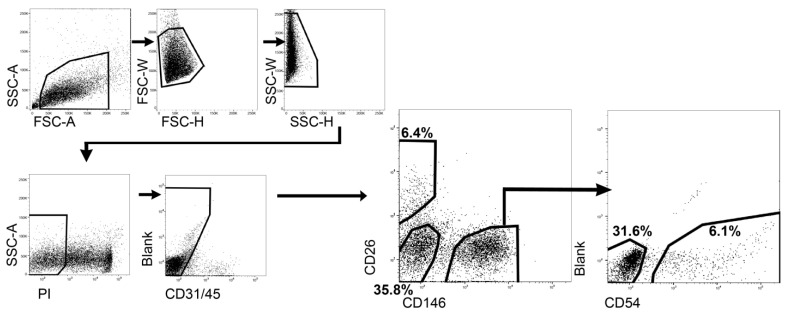
Isolation method of ASPCs based on cell surface markers. The gating strategy shown above was used for the identification of ASPCs in bovine adipose. The percentage shown on each population means the fraction of each population to whole ASPCs (CD31−CD45− live singlets).

First, we confirmed the cross-reactivity between cattle and antigen species based on previous reports [11,12,13], as the number of monoclonal antibodies targeting bovine antigens is limited. Notably, we used bovine muscle rather than bovine adipose to confirm cross-reactivity. Especially, we targeted CD29 (clone: Ha2/5) positive cells as the population had been shown to be concentrated in colony-forming cells [13]. Antigens (58 clones) were analyzed via flow cytometry and a homology search. Specifically, in flow cytometry, we evaluated “%positive (%pos.)” and “Staining index.” “%positive” is the proportion of positive events to observed events, and “Staining index” is the index commonly used to validate the degree of separation between the positive and negative population. For the homology search, we targeted the full-length amino acid sequence and calculated the p-distance after multiple sequence alignments between cattle and antigen species. Among the listed antibodies, we could pick up candidate antibodies for 15 antigens (CD49a, CD49e, CD49f, CD9, CD24, CD34, CD36, CD44, CD54, CD56, CD90, CD106, CD146, CD295, and CD344) (Supplementary Material Table S1). As a result of the comprehensive judgment, human CD146 (clone: P1H12) and mouse CD54 (clone:3E2) were selected to isolate proliferative cells from the bovine adipose tissue. In addition to the screened antibodies, we used a bovine CD26 antibody (clone: CACT114A) for the categorization of cells present in adipose tissue. We obtained SVF cells from adipose tissue around the cheek meat by mechanical and enzymatic dissociation. Lin− (CD31−CD45−) live singlets from SVF cells were analyzed via flow cytometry to determine the CD26, CD146, and CD54 expression patterns (Figure 1). Consequently, the following two points were identified. First, CD146 and CD26 were exclusively expressed. Next, most CD54+ cells (92.6% of CD54+) express CD146 and CD146+ cells were further divided based on CD54 expression. Therefore, we decided to separate ASPCs based on CD146 and CD26 expression and then to classify the CD146+ fraction into CD54+ and CD54− fractions. Finally, bovine ASPCs were classified into four fractions: CD26−CD146+CD54+ (6.1%), CD26−CD146+CD54− (31.6%), CD26−CD146− (35.8%), and CD26+CD146− (6.4%) (Figure 1).

### 2.2. Cell Morphology and Self-Renewal Capacity of Each ASPC Population

**Figure 2:**
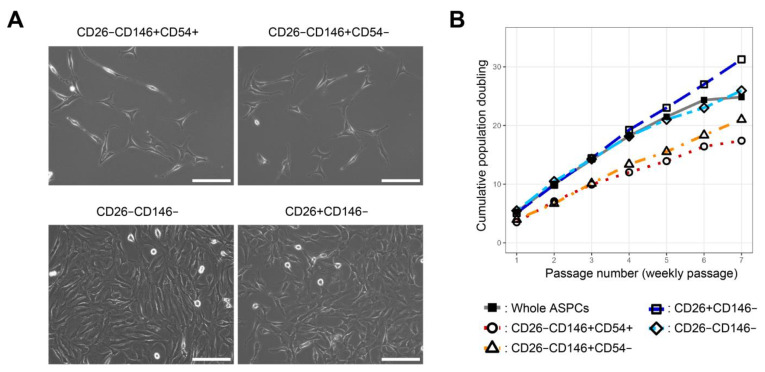
Cell morphology and growth capacity of each ASPC. (**A**) Representative cell morphology scanned via phase contrast microscopy on day 6 of primary culture (bar = 150 µm). (**B**) The proliferative capacity of each population was evaluated as cumulative population doubling (CPD). The cells were passaged every week, confirming that they were not confluent. Populations are indicated by the following symbols and color (whole ASPCs: close square and gray, CD26−CD146+CD54+: open circle and red, CD26−CD146+CD54−: open triangle and orange, CD26+CD146−: open square and blue, and CD26−CD146-: open rhombus and light blue), *n* = 1.

In primary cultures, CD26−CD146+CD54+, CD26−CD146+CD54−, CD26−CD146−, and CD26+CD146− cell populations could adhere to plastic culture dishes, which is one of the MSC criteria [14]. Two fractions of CD26−CD146+ cells adhered more slowly and were more elongated than CD26+CD146− and CD26−CD146− cells (Figure 2A). Regarding proliferative capacity, cumulative population doubling (CPD) was evaluated for each fraction. CD26−146+, particularly the CD54+ population, grew slowly (Figure 2B). In contrast, the CD26+CD146− population grew fast and was approximately 10,000 times between CD26−CD146+CD54+ and CD26+CD146− in terms of cell number for 7 weeks (Figure 2B).

### 2.3. Adipogenic Capacity of Each ASPC Population

**Figure 3:**
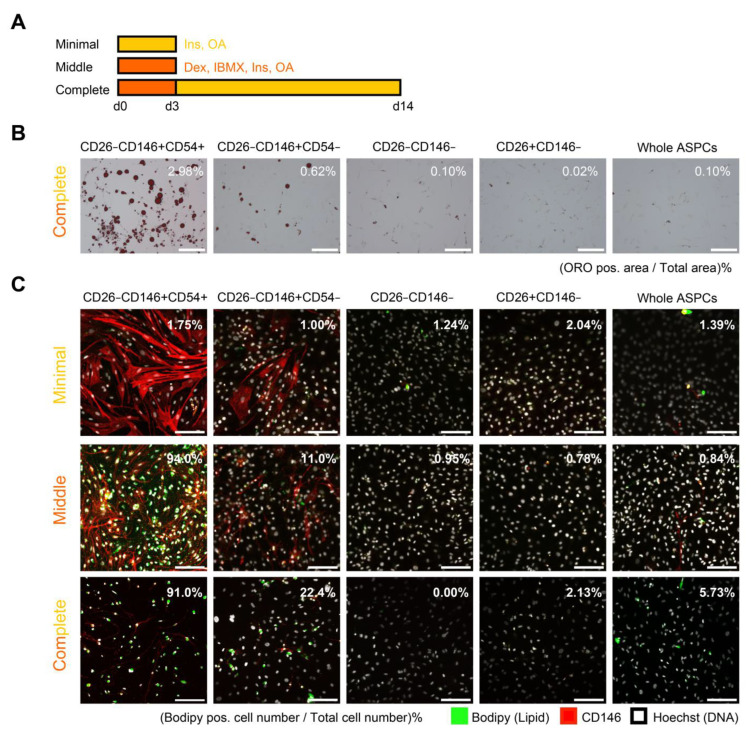
Adipogenic capacity of each ASPC population. (**A**) Schema of adipogenic induction by three methods: minimal, middle, and complete induction. Ins, insulin; OA, oleic acid; Dex, dexamethasone; IBMX, isobutylmethylxanthine. (**B**) Oil red O staining after complete induction. Bright-field images are shown (bar = 150 µm). The proportions of ORO positive areas to the total area are shown in the upper right of each image. (**C**) Immunocytochemical images of each population induced by three methods (bar = 150 µm) for Bodipy (green) and CD146 (red). Bodipy positive rates are shown at the upper right of each image.

The adipogenic capacity of each population was evaluated in vitro. First, complete induction shown in the scheme (Figure 3A), which is common for MSC induction [15], was performed for each population and cells were stained with Oil Red O (ORO) (Figure 3B). Then, CD26−146+, especially the CD54+ population, showed a high adipogenic capacity. For CD26−CD146+CD54+ cells, the ORO positive area was approximately 30 times larger than that of whole ASPCs. In contrast, CD26−CD146− and CD26+CD146− rarely showed adipogenesis. Moreover, in immunocytochemistry (ICC), CD26−CD146+CD54+ cells showed a high adipogenic capacity, and 91.0% of cells in them resulted as Bodipy positive after complete induction (Figure 3C).

Next, adipogenesis was comparatively observed after minimal, middle, and complete induction (Figure 3A,C) to confirm whether minimal stimulation could induce adipogenesis like human and mouse PreAs [6]. In brief, compared to complete induction for 2 weeks, the others were performed for 3 d only, and minimal induction lacked dexamethasone and isobutylmethylxanthine. Concerning the CD26−CD146+CD54+ fraction, few Bodipy+ cells (1.75%) were observed with minimal induction, but middle induction resulted in 94.0% Bodipy+ cells, which was comparable to complete induction. These results suggested that not only insulin and oleic acid but also dexamethasone and isobutylmethylxanthine were necessary for the CD26−CD146+CD54+ population to differentiate into adipocytes.

### 2.4. Cell Surface Marker Expression of Each ASPC Population after In Vitro Culture

**Figure 4:**
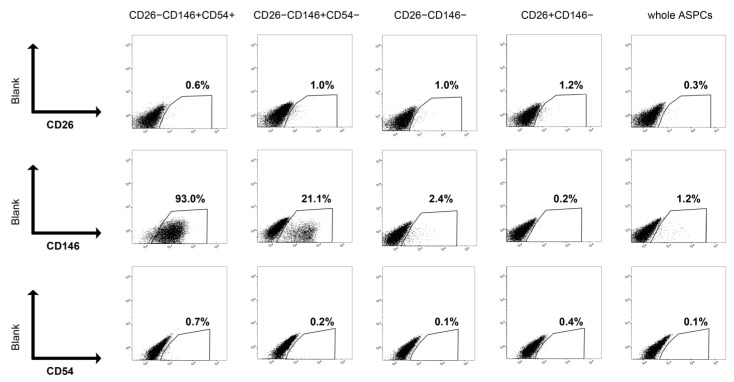
Flow cytometric analysis of cell surface markers on cultured bovine cells. Expression patterns are about the cell surface markers used for isolation. The profiles are shown for CD26−CD146+CD54+, CD26−CD146+CD54−, CD26−CD146−, CD26+CD146−, and whole ASPCs. Positive rates are shown on each plot.

Flow cytometric analyses revealed a low expression of CD26 and CD54 (Figure 4). However, we found that the two populations of CD146 positive maintained CD146 expression after culture (Figure 4). Specifically, CD146 was expressed in 93.0% of the CD26−CD146+CD54+ fraction and 21.1% of the CD26−CD146+CD54− fraction.

## 3. Discussion

This study aimed to isolate novel ASPC populations from bovine fat and confirm the conservation of markers and stemness in ASPCs, such as ASCs, PreAs, and Aregs, between cattle and other previously reported species. Our findings indicate similarities and differences, as described below.

First, possible PreAs were isolated from bovine adipose as CD26−CD146+CD54+ or CD26−CD146+CD54−. CD146, also known as a MCAM, has been reported to be a marker for highly adipogenic progenitors in adipose tissue [9,10]. Furthermore, CD54, also known as intercellular adhesion molecule-1, is a marker of PreAs in human and mouse white adipose tissue [6]. This study shared these markers; however, the fractions of positive populations were lower than those reported in previous reports. For example, concerning CD54-positive populations, there was only 6.1% in this study compared to 40.8% reported by Merrick et al. [6]. This difference probably indicates that anti-mouse CD54 (clone3E2) antibodies failed to bind to bovine CD54. Additionally, CD26−CD146+ populations, especially CD54+, from bovine fat showed slow proliferation and a high adipogenic capacity. These phenotypes are consistent with the characteristics of PreAs [1]; however, CD26−CD146+ populations from bovine fat showed lower adipogenic capacities than PreAs in humans and mice described in a previous report [6]. Specifically, possible PreAs from bovine fat cannot differentiate into adipocytes by insulin alone, but PreAs in humans and mice have been reported [6]. This difference was possibly caused by the difference in the optimal adipogenic induction method between cattle and other experimental animals. To support this possibility, adipogenic protocols for humans and mice are known to be ineffective for cattle, and three-dimensional spheroid culture and endothelial basal medium facilitate bovine adipogenesis [16].

Next, a bovine CD26-positive cell population was isolated as a candidate ASC against possible PreAs. This population comprised 6.4% of whole ASPCs (CD31−CD45− live singlets), and the percentage was comparable with a previous report [6]. In addition, the bovine CD26+CD146− cell population is highly proliferative, and this phenotype is characteristic of ASCs [1]. In contrast, bovine CD26+CD146− cells did not differentiate into adipocytes after complete adipogenic induction for 2 weeks. The human CD26-positive cell population has adipogenic capacity via complete induction. The optimization of adipogenic induction protocol for bovine CD26-positive cells may overcome this difference. Alternatively, the bovine CD26-positive population may not have an adipogenic capacity. To confirm the possibilities, it is required to try other induction methods optimized for cattle and other ruminants, such as the addition of PPAR-γ agonist [17,18] and the removal of dexamethasone and isobutylmethylxanthine [19].

We were unable to isolate two fractions from the bovine fat: CD142+ adipogenesis regulators (Aregs) and CD26+CD146+ (double positive, DP). Aregs are commonly recognized as adipogenesis suppressors in a paracrine manner; however, there is no consensus on whether Aregs can differentiate into adipocytes [5,6]. In this study, we could not use markers such as CD142 or substitutes for CD142; therefore, populations other than CD54 positive or CD26 positive were candidates for Aregs. However, CD26−CD146+CD54− or CD26−CD146− were still heterogeneous in this study. Further classification and assays are required to confirm the presence of Aregs, with a focus on adipogenesis suppression. In addition, DP was reported as the transition state between ASCs and PreAs in vivo [6]; however, we did not observe DP in prospective isolation or after in vitro culture. These observations are probably due to the poverty caused by cross-reactivity. Another possibility is that bovine CD26 positive and CD54 positive are highly independent.

In this study, we could not observe each population repeatedly and multilaterally. The position of bovine fat was in bovine cheek meat only and the biological replicate was poor. Furthermore, gene-level analyses and histochemical analyses were not carried out.

In the future, each ASPC fraction of this study can make a contribution to the evaluation of adipogenesis and lipogenesis in vitro regarding a variety of species. For example, CD26−CD146+ will be useful to validate adipogenesis and lipogenesis more accurately, as the fraction is homogeneous and high adipogenic progenitors. Furthermore, the fraction may facilitate the analysis of the fatty-acid metabolism in vitro, which is helpful in developing the livestock industry. Also, the sorting strategy and characteristics of bovine ASPCs are expected to be applied to other livestock animals as they are more closely related to cattle than humans and mice, which traces back to *Boreoeutheria*. For example, in order of closest relatives, bison and buffalo of a common tribe (*Bovini*), goats and sheep of a common family (*Bovidae*), and pigs of a common order (*Cetartiodactyla*) may share the characteristics of ASPCs in this study. ASPCs of other livestock animals can make the in vitro assay described above more comprehensive and acceptable to different cultures in terms of ethics, religion, and environment.

## 4. Materials and Methods

### 4.1. Preparation of SVF Cells from Livestock Tissue

Bovine fat was derived from bovine cheek meat after slaughter. The meat was obtained from Tokyo Shibaura Zoki Co., Ltd. (Tokyo, Japan) and transported to our laboratory on ice. Fat around the fascia of the cheek meat was harvested and stored in Hanks’ Balanced Salt Solution (HBSS) with a penicillin–streptomycin–amphotericin B Suspension (FUJIFILM Wako Pure Chemical Corporation, Osaka, Japan; 161-23181) at 4 °C until the following procedures were conducted.

SVF cells were prepared from bovine fat via mechanical and enzymatic dissociation as described previously [13]. Briefly, harvested tissues were mechanically dissected with scissors for approximately 10 min and digested with a collagenase solution. The collagenase solution consisted of 2 mg/mL collagenase (FUJIFILM; 032-22364), 25 Units/mL DNase 1 (Sigma-Aldrich, St. Louis, MO, USA; D5025), HEPES 10 mM (Nacalai Tesque, Kyoto, Japan; 17557-94), and penicillin–streptomycin–amphotericin B Suspension in Dulbecco’s modified Eagle medium (DMEM) (Gibco; 10567-014). Tissues cut into tiny pieces were rotated in collagenase solution for 1 h at 37 °C. After rotation, the tissue fragments were removed using a drain net for the kitchen, and the filtrate was centrifuged at 490× *g* for 5 min in HBSS. The cell pellet was resuspended in HBSS and centrifuged at 490× *g* for 5 min. Finally, the cell pellet was frozen in CELLBANKER 1plus (Nippon Zenyaku Kogyo Co., Ltd., Fukushima, Japan; 11912). All procedures until cell freezing were performed within 48 h of slaughter, and the cells were stored in liquid nitrogen.

### 4.2. Isolation of Each ASPC Population with Flow Cytometry 

The cells were rapidly thawed and filtered in HBSS through a 100 µm cell strainer (Corning, Durham, NC, USA; 352360). Then, the filtrate was centrifuged at 490× *g* for 5 min and resuspended in basic staining buffer, which comprised 1 mM ethylenediaminetetraacetic acid (EDTA) (Invitrogen, Carlsbad, CA, USA; 15575-038), 10 mM HEPES, 2% fetal bovine serum (FBS) (Gibco; 10270-106), and a penicillin–streptomycin–amphotericin B Suspension in HBSS. Finally, the cell suspensions were stained with the following antibodies for analysis and sorting: The primary antibody set was fluorescein isothiocyanate (FITC)-conjugated anti-sheep CD31 (clone: CO.3E1D4) antibody (1:50; Bio-Rad Laboratories, Inc., Hercules, CA, USA; MCA1097F), FITC-conjugated anti-sheep CD45 (clone:1.11.32) antibody (1:200; Bio-Rad; MCA2220F), phycoerythrin (PE)-conjugated anti-human CD146 (clone: P1H12) antibody (1:200; BD Biosciences, Franklin Lakes, NJ, USA; 550315), non-conjugated anti-bovine CD26 (clone: CACT114A) antibody (1:200; Monoclonal Antibody Center, Washington State University, Pullman, WA, USA; BOV2078), and Brilliant Violet 510 (BV510)-conjugated anti-mouse CD54 (clone:3E2) antibody (1:200; BD Biosciences; 563628). The secondary antibody was Alexa Fluor 647-conjugated anti-mouse IgG2b antibody (1:1000; Jackson ImmunoResearch Inc., West Grove, PA, USA; 115-605-207) and binding anti-CD26 antibody. The cell suspension was incubated with primary and secondary antibodies on ice for 30 min each. The cell suspension was washed once after the primary response. Finally, a 200 ng/mL propidium iodide (PI) solution (Sigma-Aldrich; P4864) was used to remove the dead cells. Flow cytometry and cell sorting were performed using an FACSAria II Cell Sorter (BD Biosciences).

### 4.3. Cell Culture, Proliferation Assay, and Cell Surface Marker Analysis

Isolated cells were cultured on plastic dishes (Thermo Fisher Scientific Inc., Waltham, MA, USA; 150464, 150468) in DMEM (Gibco; 10567-014), which contained 10% FBS, 10 mM HEPES, and penicillin–streptomycin–amphotericin B Suspension in 5% CO_2_ at 37 °C. The medium was changed twice weekly and cultured cells were passaged with 0.05% trypsin-EDTA (Gibco; 25300-620) until confluence. Proliferation assays were performed from passages 1 to 7, and CPD was evaluated. The number of passaged cells was accordingly adjusted not to cause contact inhibition.

For cell surface marker analysis, the cultured cells were detached using trypsin-EDTA. The cells were then stained with antibodies and analyzed via flow cytometry in the same manner as for cell isolation.

### 4.4. Adipogenic Differentiation

Adipogenic differentiation was assessed via ORO staining and ICC analysis for each population at early passages. The three methods used for adipogenic differentiation were complete, middle, and minimal induction. In complete induction, DMEM (Gibco; 10567-014) was supplemented with 1 µg/mL of human recombinant insulin (FUJIFILM; 093-06471), 400 µM of oleic acid (Tokyo Chemical Industry Co., Ltd., Tokyo, Japan; O0180) conjugated with bovine serum albumin (Nacalai Tesque; 19361-84), as previously described [20], and penicillin–streptomycin–amphotericin B suspension. Furthermore, 1 µM dexamethasone (FUJIFILM; 041-18861) and 500 µM isobutylmethylxanthine (FUJIFILM; 095-03413) were added to the induction medium for the first 3 d. Complete induction was performed over 2 weeks. In contrast, middle and minimal inductions were performed for 3 d. The middle induction was identical to the first 3 d of complete induction, and the minimal induction lacked dexamethasone and isobutylmethylxanthine. Each fraction was passaged on laminin (FUJIFILM; 120-05751)-coated plastic plates (Greiner Bio-One, Kremsmünster, Oberösterreich, Austria; 665180) for ORO or glass chambers (Matsunami Glass Ind., Ltd., Osaka, Japan; SCS-N08) for ICC and induced after confluency. 

### 4.5. ORO Staining

Induced cells were fixed in 4% paraformaldehyde (FUJIFILM, 163-20145) for 10 min and washed with phosphate-buffered saline (PBS) twice. Then, the cells were acclimated with 60% 2-propanol (Nacalai Tesque; 29112-63) for 3 min and stained with ORO working solution for 10–20 min, which comprised ORO solution (Muto Pure Chemicals Co., Ltd., Tokyo, Japan; 40491) and water in the ratio of 6:4. After washing with 60% 2-propanol for a short period, the cells were further washed with water thrice. Stained cells were observed using a BZ-X710 microscope (Osaka, Japan). Scanned images were then analyzed to evaluate the ORO positive area using ImageJ 1.53q software (Supplementary Material Figure S1) [21,22].

### 4.6. ICC

Induced cells were fixed, washed as described above, and blocked with 2% bovine serum albumin (BSA) in PBS for 1 h at room temperature. Then, the cells were stained with the following primary antibody in 2% BSA/PBS overnight at 4 °C: PE-conjugated anti-human CD146 (clone: P1H12) antibody (1:100; BD Biosciences; 550315). After two washes, the cells were stained with Hoechst 33258 (1:1000; FUJIFILM; 343-07961), 1 µg/mL Bodipy 493/503 (Invitrogen; D3922), and Alexa Fluor 555-conjugated anti-mouse IgG1 secondary Antibody (1:1000; Invitrogen; A-21127) for 1 h at room temperature. The stained cells were washed twice and mounted using Fluoremount g (Southern Biotech, Birmingham, AL, USA; 0100-01). The prepared samples were observed under an LSM 700 confocal microscope (Zeiss, Oberkochen, Germany).

### 4.7. Confirmation of Cross-Reactivity between Bovine and Other Species for Immunogen

Thirty antigens (58 clones) were analyzed using flow cytometry and a homology search. For flow cytometry analyses, targeted SVF cells were isolated from bovine muscle and analyzed using a FACSAria II cell sorter (BD Biosciences). SVF cells were pre-gated as CD29 (Ha2/5)-positive singlets (BD Biosciences; 562153 or 562154) to concentrate colony-forming cells, as described previously [13]. Then, the cells were validated for reactivity of each antibody in terms of “%pos” and “Staining index.” “%pos” was defined as the fraction of positive events to total CD29+ singlets. Additionally, the “Staining index” was commonly defined as the formula below, showing the degree of separation between the positive and the negative populations.

{MFI (pos.) − MFI (neg.)}/2 × SD (neg.)

MFI: Mean Fluorescence Intensity, SD: Standard Deviation

In the homology search, the p-distance was measured as the evolutionary distance between the cattle and antigen species. The amino acid sequence of each protein was obtained from the National Center for Biotechnology Information. Notably, the information on the focal site targeted by each antibody was often close to the public; therefore, we analyzed the full length of the total amino acids. Multiple sequence alignment (ClustalW) and measurements of the p-distance by pairwise deletion were performed using the Molecular Evolutionary Genetics Analysis software [23,24,25].

## 5. Conclusions

Our results showed similarities and differences in both markers and stemness between cattle and other species, suggesting that collecting and characterizing possible bovine PreAs as CD26−CD146+CD54+ or CD26−CD146+CD54− is possible. More detailed analyses of gene expression and histochemical locations will help clarify these characteristics more accurately. In addition, we were unable to characterize possible bovine ASCs or collect bovine Aregs. Optimized induction protocols for ASCs and novel Areg markers for cattle will be required in the future.

## Data Availability

The datasets used in this study are available from the corresponding author upon request.

## Supplementary Materials

The following supporting information can be downloaded from https://www.mdpi.com/article/10.3390/ijms241511908/s1.
