# Supplementary material for: Conservation of Markers and Stemness in Adipose Stem and Progenitor Cells between Cattle and Other Species
Source: Int J Mol Sci. 2023 Jul 25;24(15):11908. doi: 10.3390/ijms241511908 (PMC10418360; doi:10.3390/ijms241511908)
Supplement: Supplementary file 1 [file ijms-24-11908-s001.zip › ijms-2497792-supplementary.pdf]

Low-power field (scale bar = 2000 μm)

Pre-analysis

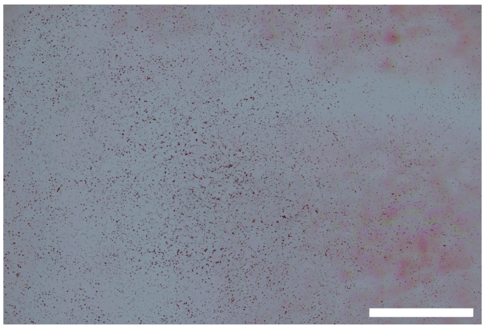

Setting color threshold and binarization

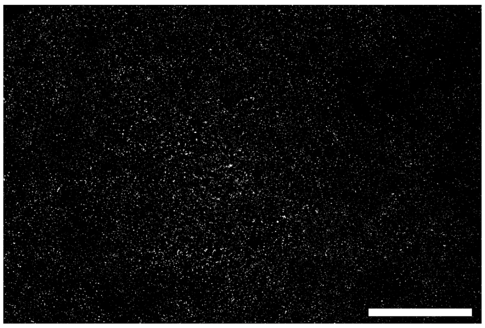

Particle analysis

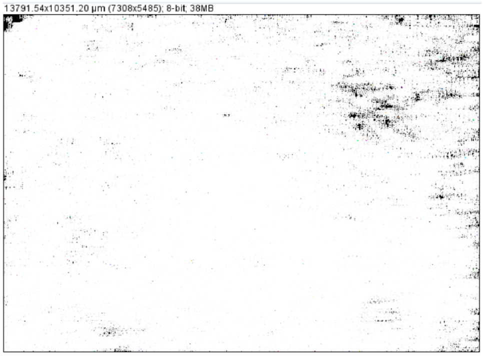

ORO-positive area 2.98%
Total particle number 29611

High-power field (scale bar = 300 μm)

Pre-analysis

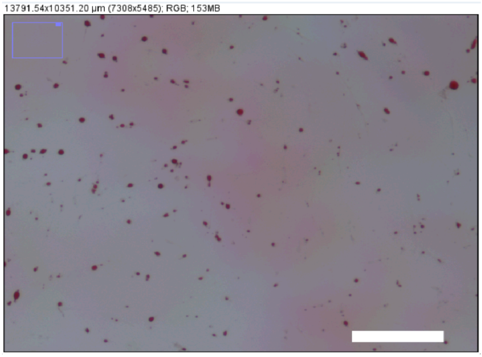

Setting color threshold and binarization

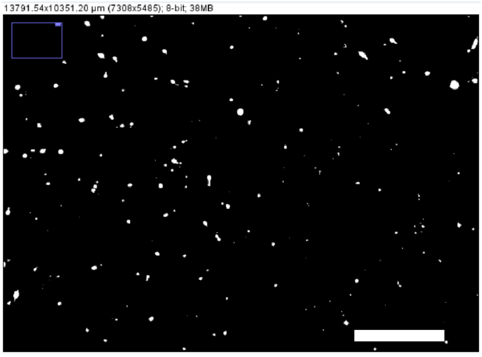

Particle analysis

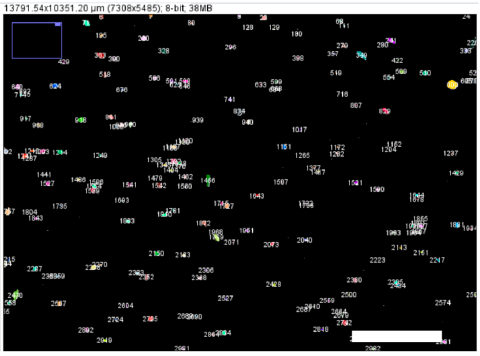

Supplementary Material Figure S1. Quantification of ORO-positive area
Quantification of ORO-positive area was performed using ImageJ software. The images examplified above were CD26−CD146+CD54+ to differentiate into adipocytes by complete induction. The images were processed by the algorithm of color thresshold and binarization. Finally, ORO-positive area was evaluated by particle analysis.

Supplementary Material Table S1. Cross-reactivity between cattle and antigen species

Cell surface markers	Immunogen	Clone	%pos	Staining index	P-distance
CD49a (ITGA1)	human	SR84	26.9	4.4E+05	0.09
	rat/mouse	Ha31/8	0.4	1.6E+05	0.12/0.12
CD49b (ITGA2)	human	12F1	0.0	–	0.14
	rat	Ha1/29	0.3	1.8E+05	0.20
	mouse	HM α2	0.2	1.0E+05	0.21
CD49c (ITGA3)	human	C3 II.1	0.0	–	0.10
CD49d (ITGA4)	human	9F10	0.1	3.5E+04	0.16
	rat	MRα4-1	0.0	–	0.18
	mouse	R1-2	0.5	1.4E+05	0.18
CD49e (ITGA5)	human	IIA1	2.9	6.7E+05	0.08
	mouse	5H10-27	0.7	1.4E+06	0.11
CD49f (ITGA6)	human	GoH3	33.1	1.4E+06	0.08
integrin a-7 (ITGA7)	mouse	3C12	0.7	3.4E+04	0.15
CD51 (ITGAV)	mouse	RMV-7	0.2	6.7E+05	0.08
CD104 (ITGB4)	human	439-9B	0.0	–	0.11
CD9	human	M-L13	0.0	–	0.23
	mouse	KMC8	7.9	9.4E+06	0.16
	mouse	MZ3	0.9	1.7E+06	
CD24	rat	HIS50	2.3	2.8E+05	0.34
CD26	rat	OX-61	0.4	1.6E+05	0.17
CD34	human	581	0.0	–	0.41
	mouse	HM34	3.6	7.0E+04	0.47
	mouse	RAM34	0.8	8.2E+04	
CD36	mouse	CRF D-2712	7.0	4.6E+05	0.16
CD44	human	G44-26	0.0	–	0.35
	human	515	0.8	1.1E+05	
	mouse	IM7	10.3	2.2E+06	0.41
CD44H	rat	OX49	0.2	2.1E+05	0.35
CD54	rat	1A29	0.1	5.9E+04	0.49
	mouse	3E2	2.9	3.1E+05	0.47
CD56	human	MEM188	3.3	9.8E+05	0.04
	human	B159	0.0	–	
	human	NCAM16.2	2.7	2.7E+05	
CD73	human	AD2	0.0	–	0.10
	human	REA804	0.6	2.0E+05	
	mouse	TY/23	0.0	–	0.15
	mouse	TY/11.8	0.2	8.9E+04	
CD90	human	5E10	6.0	1.4E+06	0.27
	rat	OX-7	0.0	–	0.31
CD90.2	mouse	53-2.1	0.3	4.5E+04	0.31
	mouse	30-H12	0.0	3.8E+05	
CD105	human	266	0.1	9.4E+04	0.30
	mouse	MJ7/18	0.1	3.1E+04	0.35
CD106	human	STA	0.0	3.3E+04	0.25
	human	51-10C9	0.0	–	
	rat	MR106	7.5	1.6E+05	0.29
	mouse	429	0.1	–	0.31
CD140a	human	aR1	0.0	–	0.08
	human	16A1	0.1	1.1E+05	
	mouse	APA5	0.9	1.1E+05	0.12
CD146	human	P1H12	13.8	1.1E+06	0.46
	mouse	ME-9F1	1.1	3.4E+05	0.55
CD220	human	3B6/IR	0.0	–	0.04
CD295	human	52263	5.3	1.2E+05	0.17
CD344	human	CH3A4A7	12.1	3.2E+05	0.02
Sca-1	mouse	D7	0.1	6.3E+04	–
	mouse	E13-161.7	0.9	8.0E+04	
AP	human	B4-78	0.0	–	0.11

Red letters indicate any of the following conditions below.

1. % pos >= 1.0
2. Staining index >= 4.0 x 10^5
3. p-distance <= 0.1

Notes

%pos = positive events / observed CD29 pos. singlets, by flow cytometry
Staining index = {MFI (pos.) - MFI (neg.)} / 2 x SD (neg.), to validate the separation between positive population and negative population
(MFI: Mean Fluorescence Intensity, SD: Standard Deviation)
P-distance (Full-length amino acid sequence)
Acquiring amino acid sequences by NCBI data base
Multiple sequence alignment between cattle and antigen species by ClustalW (supported by DDBJ)
Measurement of p-distance as evolutionary distance in the manner of pairwise deletion by MEGA (Molecular Evolutionary Genetics Analysis) software
